# Cu–Cu Grain Intermixing Quantification for Hybrid Bonding Applications

**DOI:** 10.3390/nano16211424

**Published:** 2026-11-05

**Authors:** Ankit Kumar, Jeyavel Velmurugan, Jae Shin, Roshni Ramakrishnan, Bryan Buckalew

**Affiliations:** Lam Research Corporation, 11155 SW Leveton Drive, Tualatin, OR 97062, USA

**Keywords:** Cu–Cu hybrid bonding, grain intermixing, advanced packaging, FIB-SEM, computer vision, fine-grain copper, low-temperature bonding

## Abstract

Cu–Cu hybrid bonding at reduced thermal budgets depends critically on reliable grain intermixing across the bonding interface. Yet interfacial evolution is still predominantly assessed qualitatively, precluding objective comparison across copper microstructures and process conditions. Here, we introduce Bond IQ, an automated image-based metrology framework that converts registered cross-sectional FIB-SEM datasets into physically interpretable descriptors using classical computer-vision methods. We apply the framework to five blanket chip-level pairings spanning three engineered copper microstructures: coarse-grain (CG), fine-grain locked (FG-L), and fine-grain thermally dynamic (FG-TD) Cu, thermocompression bonded at 210 °C for 2 h. Three coupled but distinct metrics are extracted: Bridged Interface Fraction (BIF), Interface Void Loading (IVL), and normalized Grain Penetration Depth (GPD). The symmetric CG/CG interface exhibited the weakest evolution, combining high IVL (5.4 nm) with low BIF (6.1%) and low GPD (0.20). By contrast, CG/FG-TD achieved the highest BIF (54.5%) at low IVL (0.6 nm), signifying broad but comparatively shallow bridging, whereas CG/FG-L reached the highest GPD (0.89) at moderate BIF (32.7%), indicating localized yet deeper directional grain advancement. Among symmetric fine-grained interfaces, FG-L/FG-L displayed the lowest IVL (0.5 nm), underscoring the role of fine-grain stability in void suppression. Collectively, these results demonstrate that interface bridging, void elimination, and penetration depth are governed by distinct but interrelated contributions of grain-size contrast and microstructural stability. By reducing subjective interfacial assessment to quantitative, mechanistically interpretable descriptors, Bond IQ offers a scalable route to screen copper microstructures and bonding conditions, and it can be extended to other metal–metal interfaces where microstructural evolution governs low-temperature bonding reliability.

## 1. Introduction

Hybrid bonding has emerged as a key interconnect technology for advanced semiconductor packaging, enabling direct Cu–Cu electrical connection together with dielectric bonding at fine pitch for high-bandwidth memory, logic-memory stacking, and heterogeneous 3D integration [1,2,3,4,5,6], with wafer-to-wafer interconnect pitches demonstrated down to 400 nm [7]. Hybrid bonding refers to the direct bonding of two wafers or dies in which the dielectric surfaces bond to each other while embedded Cu pads on the opposing surfaces bond simultaneously, forming the electrical connections without solder or adhesive. The Cu–Cu bond is typically formed by thermocompression bonding, in which heat and pressure are applied together. In the present study, blanket Cu films without dielectric were bonded, so only the Cu–Cu component of hybrid bonding is examined. Compared with conventional solder-based interconnect approaches, Cu–Cu hybrid bonding offers advantages in pitch scaling, parasitic resistance, thermal budget, and integration density, and is therefore increasingly important for next-generation device architectures [1,2,3,4]. However, successful bond formation depends strongly on the microstructural evolution of the Cu–Cu interface, which is influenced by grain structure, surface planarity, oxide removal, alignment, and post-bond thermal treatment [1,2,8,9,10]. These factors collectively determine whether the bonded interface evolves toward a continuous metallurgical joint or remains interrupted by residual voids and incomplete grain coalescence.

At the Cu–Cu bonding interface, metallurgical bond development is governed by grain boundary migration, grain growth across the original interface, local elimination of the bond line, and progressive suppression of interfacial voids [2,11,12,13,14,15,16] as shown in Figure 1. Here, grain intermixing denotes the growth or migration of Cu grains across the original interface so that grains of the top and bottom Cu layers interpenetrate and the bond line is progressively replaced by continuous Cu. Recent studies have shown that engineered fine-grain or nanocrystalline Cu can promote cross-interface grain growth and enable stronger bonding under lower thermal budgets, whereas coarse-grain Cu microstructures often require more aggressive annealing to achieve comparable intermixing [11,12,13,14,17]. These findings indicate that the bonded interface should not be described simply as bonded or unbonded; rather, it should be assessed in terms of the extent, continuity, and morphology of microstructural evolution across the interface. As a result, the initial microstructure pairing between the top and bottom Cu surfaces (here, top and bottom refer to the Cu films on the upper and lower Cu-coated pieces of the bonded pair, respectively, as oriented in the cross-sectional images) is expected to play a central role in determining bonding response. In addition to such microstructure engineering, other low-temperature routes act on the surface rather than on the Cu grain structure, for example, plasma activation to obtain an oxide-free Cu surface [9] and metal passivation layers whose crystallinity governs interfacial diffusion [18].

Bond formation under thermocompression involves several overlapping, thermally activated processes. First, the applied pressure deforms the surface asperities plastically and by creep, which increases the real contact area without macroscopic deformation of the bonded stack. Second, atomic diffusion along grain boundaries and interfaces, which is the dominant mass-transport path at these low temperatures, moves Cu into the residual interfacial voids and progressively shrinks and closes them [22,23]. Third, grain boundaries that intersect the interface migrate, driven by the reduction in grain-boundary and interfacial energy (and by stored strain energy in fine-grained Cu), which promotes grain growth across the original interface and the progressive disappearance of the bond line [11,12,13,14,15,16,24]. The final bond quality depends on the balance between densification by pore closure and grain growth. If boundaries migrate faster than pores can be removed, pores can become isolated inside grains, where only slow lattice diffusion can remove them; efficient pore removal while pores are still attached to boundaries instead gives a more homogeneous and continuous joint [23,25]. Residual interfacial pores can also pin the migrating boundaries (Zener-type pinning), because separating a boundary from a pore would increase the total grain-boundary area, and this pinning force restrains local grain growth and cross-interface migration [25,26]. Interfacial porosity and grain evolution are therefore coupled, which is why Bond IQ, introduced below, reports interface bridging, void loading, and grain penetration as separate descriptors.

The initial gap between the surfaces in Figure 1 is not closed by thermal expansion alone. As a first-order estimate, heating a 2.5 µm Cu film from room temperature to 210 °C (ΔT ≈ 185 K, thermal expansion coefficient ≈ 17 × 10^−6^ K^−1^) expands it by about 8 nm in the out-of-plane direction if it is unconstrained (constraint by the Si substrate changes this value but not its order of magnitude), i.e., roughly 16 nm for the two films. Thermal expansion can therefore help close nanometer-scale gaps, but it is unlikely to bring the surfaces into atomic contact over the full area by itself. The applied pressure is an essential contributor: because the load is initially carried by the tips of the surface asperities, the local contact stress is much higher than the nominal pressure and drives plastic deformation and creep of the asperities, which brings the surfaces into the atomic-contact range where diffusion-driven void closure can proceed [22]. The separate contributions of thermal expansion and pressure were not measured in this study. Figure 1 therefore shows the applied bonding pressure together with temperature as the conditions that close the gap.

Despite this recognized importance, Cu–Cu intermixing is still commonly described using qualitative terms such as grain regrowth, interface disappearance, zig-zag interface, or improved bonding [15,16]. Although these descriptors are useful for visual interpretation, they do not provide a standardized numerical basis for comparing process conditions, electrolyte chemistries, or dissimilar top/bottom microstructure pairings. High-resolution techniques such as transmission electron microscopy (TEM) can reveal local structural details, but they are costly, destructive, low-throughput, and often restricted to very small fields of view. In contrast, focused ion beam–scanning electron microscopy (FIB-SEM) provides a more practical route for process development, yet its interpretation is still frequently manual and therefore susceptible to operator bias, especially when comparing interfaces formed from dissimilar Cu grain structures [2,3,12]. This metrology gap limits systematic establishment of process–microstructure–bonding relationships and slows optimization of low-temperature hybrid bonding technologies.

To address this gap, we investigated five thermocompression-bonded blanket Cu-film combinations, derived from three different plating processes, to establish how the initial top and bottom microstructure pairing governs post-bond intermixing under a common thermal budget. The combinations span three microstructural states relevant to hybrid bonding: coarse-grain (CG) Cu, which retains a coarse structure both before and after bonding; fine-grain locked (FG-L) Cu, which preserves its fine grain structure through the bonding anneal; and fine-grain thermally dynamic (FG-TD) Cu, which is fine-grained as-deposited but recrystallizes and coarsens upon reaching the bonding temperature. Pairing these states yields both symmetric interfaces, formed between identical microstructures, and asymmetric interfaces, formed between dissimilar ones, representative of the pad-to-pad combinations encountered in hybrid bonding process development.

To replace qualitative interface assessment with a standardized numerical basis, we developed an automated, image-based framework comprising Grain IQ and Bond IQ. Operating directly on cross-sectional FIB-SEM images, Grain IQ segments the Cu microstructure using classical computer-vision methods, and Bond IQ extracts three physically interpretable, grain-size-normalized descriptors of the bond line: the Bridged Interface Fraction (BIF), quantifying the extent to which the interface has closed into continuous Cu; the Interface Void Loading (IVL), quantifying residual voiding retained along the bond line; and the Grain Penetration Depth (GPD), quantifying cross-interface grain advancement. Together, these descriptors capture interface continuity, void suppression, and grain intermixing within a single reproducible framework, providing an objective basis for comparing bonding response across dissimilar Cu microstructures and mitigating the operator bias inherent to manual FIB-SEM interpretation. In subsequent work, these descriptors can be correlated with measured mechanical bond strength and weighted to construct a composite intermixing index that more directly reflects overall interfacial quality.

The main contribution of Bond IQ relative to previously reported characterization and image-analysis methods is therefore not automated grain segmentation alone. It is the combination of (i) registered secondary-electron and ion-channeling FIB-SEM datasets, in which the first channel locates voids and the bond line and the second resolves grains, with (ii) three separate, grain-size-normalized interface descriptors that distinguish interface bridging, void retention, and penetration depth. This combination allows dissimilar top/bottom Cu microstructure pairings to be compared quantitatively over approximately 100 µm of interface, which manual assessments or single-parameter metrics such as bonded or void fractions [27] do not provide.

## 2. Materials and Methods

### 2.1. Sample Preparation and Cu–Cu Bonding

Blanket Cu films with a thickness of 2.5 µm were electroplated onto metallized Si wafers (300 mm in diameter and 775 µm thick, supplied in-house at Lam Research) with a 100 Å Ta barrier layer and an 800 Å Cu seed layer deposited by physical vapor deposition (PVD). The Cu was therefore electroplated onto the PVD Cu seed layer rather than directly onto Ta (layer stack: Si/Ta/Cu seed/electroplated Cu), because the high resistivity of Ta precludes direct electroplating [28]. Ta is a standard diffusion barrier for Cu [28,29,30], and the two metals are immiscible and form no intermetallic compounds [31]. Here, the Ta layer is separated from the Cu–Cu bond interface by about 2.5 µm of Cu on each side, and the pre-bond anneal (100 °C) and bonding (210 °C) temperatures are far below the 550–600 °C up to which 50 nm Ta films have been reported to block Cu [29,30]. No significant Cu–Ta interaction is therefore expected, although this was not examined separately. Electroplating was performed using a SABRE^®^3D system (Lam Research, Tualatin, OR, USA). Three plating conditions, designated Process A, Process B, and Process C, were established using different chemistry and waveform combinations, as summarized in Table 1. These conditions produced the three distinct Cu microstructure classes evaluated in this study: CG, FG-L, and FG-TD.

Following electroplating, all wafers were subjected to a pre-bond anneal at 100 °C for 1 h, selected to remain within the targeted thermal budget. A common surface-preparation sequence consisting of chemical mechanical planarization (CMP), a polishing process that combines chemical reaction and mechanical abrasion to planarize the Cu surface and reduce its roughness, and plasma treatment [9] was subsequently applied before bonding. The Cu surfaces were additionally cleaned in 50 wt.% citric acid for 1 min, rinsed with deionized (DI) water, and purged with nitrogen (N_2_) gas before bonding. The root-mean-square surface roughness (Rq) of the Cu surfaces after CMP was less than 5 nm. Cu–Cu thermocompression bonding of chips diced from the plated wafers was performed at 210 °C for 2 h under an applied bonding pressure of 22 MPa in a vacuum ambient. The bonding conditions were selected on an empirical basis, as representative of the low-temperature thermocompression conditions commonly used in the Cu–Cu bonding literature and in industrial hybrid-bonding development [1,2,11,12]. They are also consistent with diffusion-bonding theory, in which pressure-assisted asperity deformation and temperature-activated grain-boundary diffusion govern void closure [22]. Bonding was performed at National Yang Ming Chiao Tung University on a die-to-die bonder (CA-2000VA, Bondtech Co., Ltd., Kyoto, Japan; alignment accuracy ±1 µm). Chips diced from the plated wafers were aligned and bonded in vacuum, with the load applied by the bonder while the sample was heated to and held at 210 °C. The top chips (6 × 6 mm^2^) were smaller than the bottom chips (8 × 8 mm^2^), so that the bonded area remains constant even when there is some misalignment during bonding. Because the nominal pressure of 22 MPa is far below the compressive strength of Si, and the load is applied over the full area of the smaller (6 × 6 mm^2^) top chip, chip fracture, which in brittle Si typically originates from non-uniform loading, particles, or edge defects, is not expected, and no chip cracking or damage was observed after bonding. All other bonding conditions were held constant across all sample combinations, enabling differences in interfacial evolution to be attributed primarily to the initial Cu microstructure pairing rather than to variations in bonding thermal history.

Five bonded combinations were selected to represent symmetric and asymmetric top/bottom Cu microstructure pairings (see Section 3.2). Representative plan-view microstructures before and after thermal treatment on blanket wafers are shown in Figure 2. The CG condition retained a coarse-grained microstructure throughout the thermal treatments. Both FG-L and FG-TD remained fine-grained after the intermediate anneal at 100 °C for 1 h; however, FG-L retained its fine-grained structure after the bonding thermal cycle, whereas FG-TD underwent thermally dynamic recrystallization and grain growth. For each microstructure combination, three samples were obtained from a single bonded pair, and one cross-section was prepared per sample; one of these was used for the quantitative analysis and the other two were examined qualitatively. These replicates therefore represent within-sample spatial variability rather than independent bonding-run variability. Each stitched cross-sectional dataset spanned approximately 100 µm, and the section used for the quantitative analysis was selected using the predefined criteria described in Section 3.3.

### 2.2. FIB-SEM Imaging

After Cu–Cu bonding, cross-sectional FIB-SEM characterization was performed to evaluate the bonded interface. Cross-sectioning and imaging were performed by an external vendor. Cross-sectional specimens were prepared by focused-ion-beam (FIB) milling of a trench across the bonded interface, followed by a low-current polishing cut to expose a clean cross-section. For each bonded sample, twenty sequential or overlapping secondary-electron (SE) image fields were acquired along the interface with 15% overlap between adjacent fields. Ion-channel images were subsequently acquired at the corresponding field locations using the same overlap. The SE and ion-channel image fields were independently stitched in Fiji to generate approximately 100 µm-wide cross-sectional datasets. The stitched SE and ion-channel datasets were subsequently registered (that is, spatially aligned so that each pixel of the SE image corresponds to the same physical location in the ion-channel image) using the *TurboReg* plugin in Fiji, with manual initialization followed by automatic image alignment to correct offsets between the two acquisition modes. Imaging was carried out at 30 kV and 50,000× magnification. The aligned image datasets provided complementary information on interfacial voids, intergranular contrast transitions, and grain morphology across the Cu–Cu interface and served as the direct input for subsequent analysis. Continuous acquisition provided broad interface coverage while reducing local field-of-view bias. All image pairs were analyzed using the same workflow settings for a given comparison set to preserve consistency during cross-condition evaluation.

### 2.3. Bond IQ Workflow

Grain intermixing across the bonded interface is a key microstructural indicator of successful Cu–Cu bond formation. To quantify this intermixing objectively across process-dependent bonding conditions, an automated computer-vision workflow, referred here as Bond IQ, was developed to operate directly on cross-sectional FIB-SEM images. The workflow uses two complementary image channels acquired at the same interfacial location: an SE image, which resolves interfacial voids and localizes the bond line, and an ion-channeling image, which provides intergranular contrast arising from orientation-dependent channeling of the Cu microstructure. To increase the effective region of interest and improve statistical robustness, adjacent SE and ion image fields were stitched, registered, and mutually aligned, as illustrated in Figure 3. Bond IQ supports both void-based and contrast-transition approaches for bond-line detection. In this study, the void-based method was selected: the centers of visible interfacial voids in the stitched SE image were used as reference points, a fitted bond line was interpolated through regions without visible voids, and the resulting bond-line reference was transferred to the spatially registered ion-channel image. Because the bond line in void-free regions is an interpolation between neighboring void centers rather than a directly observed feature, its position was checked only by overlaying it on the registered ion-channel image and was not independently verified. The uncertainty of the reconstructed bond line is therefore largest where visible voids are sparse, and it can affect all three descriptors (see Section 3.4).

Grain segmentation from the ion images is performed by a dedicated sub-module, Grain IQ, whose workflow is summarized in Figure 4. The analysis pipeline begins with contrast enhancement using non-local means denoising and contrast-limited adaptive histogram equalization (CLAHE), followed by grain-boundary delineation using Canny edge detection. The resulting boundaries are inverted to generate grain masks, after which distance-transform and watershed segmentation algorithms are applied to isolate individual grains and quantify the grain-size distribution. The hyperparameters for each processing step were iteratively tuned and validated by overlaying the processed output onto the original ion-channel image. This approach ensured preservation of the microstructural features of interest and maintained consistency between the segmented grain structure and the original image contrast. Parameters were adjusted until the overlaid grain boundaries followed the contrast transitions visible in the original ion image without merging neighboring grains or splitting single grains. The same processing settings were then applied to all samples, and the overlays of every sample were checked against the original images. Grain IQ was validated against electron backscatter diffraction (EBSD) by correlating the average grain sizes obtained from more than five images with the EBSD results. Grain IQ gave average grain sizes lower than EBSD by 3–20 nm in absolute terms, or approximately 1.2–7.4% in relative terms. The EBSD maps were 500 × 500 pixels at 20 nm per pixel (10 × 10 µm). Figure 5 presents one representative comparison from the same specimen and processing condition, for which Grain IQ measured an average grain size of 248 nm compared with 251 nm from EBSD (a difference of about 1.2%).

With the bond line localized, interfacial voids segmented from the SE image by Otsu thresholding, and grains segmented from the ion image, a set of physics-based descriptors was defined to quantify Cu–Cu grain intermixing. Rather than combining these into a single composite score, each is reported independently to preserve information associated with distinct aspects of interfacial microstructural evolution during Cu–Cu bond formation.

Bridged Interface Fraction (BIF). BIF quantifies the extent of grain bridging across the original Cu–Cu interface and serves as a direct measure of interface elimination and metallurgical continuity. It is defined as the fraction of the analyzed bond-line length occupied by grains that extend across both sides of the original interface (Equation (1); Figure 6a). A segmented grain was classified as bridging only if the cross-interface connection exhibited a minimum continuous width of 3 pixels (approximately 40 nm), thereby excluding isolated pixel connections and segmentation artifacts. Higher BIF values indicate that a greater proportion of the nominal interface has been replaced by structurally continuous Cu, reflecting more extensive metallurgical bridging.

In Equation (1), *L_i_* represents the length of the original bonding interface over which grain intercrossing or continuous Cu bridging is detected, and *L* is the total analyzed bonding-interface length. Thus, BIF is reported as the summed bridged length normalized by the total interface length.

(1) $$\mathrm{Bridge}\;\mathrm{Interface}\;\mathrm{Fraction}=\frac{\sum_{i=1}^{i=n}L_{i}}{L}$$

Interface Void Loading. IVL quantifies the residual void burden associated with the bonding interface, defined as the total interfacial void area normalized by the analyzed bond-line length (Equation (2); Figure 6b). The interfacial region of interest was defined as a 1.0 µm-high band extending ±0.5 µm from the reconstructed bond line. All segmented voids located within this band were included in the IVL calculation. Lower IVL values denote more complete interfacial consolidation, whereas higher values indicate greater retention of residual voids.

In Equation (2), *A_v,i_* represents the area of each segmented interfacial void within the defined ±0.5 µm region around the reconstructed bonding interface, and *L* denotes the total analyzed bonding-interface length. IVL therefore has units of length and represents the equivalent void area per unit interface length.

(2) $$\mathrm{Interface}\;\mathrm{Void}\;\mathrm{Loading}=\frac{\sum_{i=1}^{i=n}A_{v,i}}{L}$$

Grain Penetration Depth. GPD quantifies the depth of grain advancement across the original Cu–Cu interface and serves as a measure of interfacial microstructure integration (Equation (3); Figure 6c). For each grain identified as spanning the reconstructed bond line, the penetration distance was defined as the maximum extent of the grain that extends into the opposing Cu layer. The average grain sizes of the top and bottom Cu layers were measured directly from the corresponding bonded-interface ion-channel images using Grain IQ. For each bonded pair, the penetration distance was normalized by the larger of these two average grain sizes, defined as *D*_max_ = max ($\bar{D}$_top_, $\bar{D}$_bottom_), where $\bar{D}$_top_ and $\bar{D}$_bottom_ are the average grain sizes of the top and bottom Cu layers respectively. This normalization accounts for the tendency of larger grains to grow preferentially into finer-grained regions and reduces bias arising from asymmetric grain-size distributions across the bonded interface. Higher normalized GPD values therefore indicate greater cross-interface grain advancement relative to the dominant initial grain-size scale. The single GPD value reported for each bonded pair is the mean of the normalized penetration distances of all grains spanning the bond line (Equation (3)).

In Equation (3), *N* is the number of cross-interface grains (grains spanning the reconstructed bond line), *d_i_* represents the measured penetration distance of an individual grain across the original bonding interface into the opposing Cu layer, and *D*_max_ is the larger average grain size of the top or bottom Cu layer. The normalized GPD is therefore obtained by averaging the cross-interface penetration distances and scaling them by *D*_max_. Direction-specific (top-to-bottom and bottom-to-top) penetration values can be reported separately as supplementary outputs, but they are not the GPD value used for the comparison in Figure 7 and Section 3.3.

(3) $$\mathrm{GPD}=\frac{1}{N}\sum_{i=1}^{N}\frac{d_{i}}{D_{\max}}$$

In this work, comparative bonding behavior is evaluated directly through BIF, IVL, and GPD rather than a single aggregated index. Preserving the individual descriptor trends provides clearer physical insight into how distinct Cu microstructure pairings govern interface continuity, void retention, and grain advancement under a common bonding condition.

## 3. Results and Discussion

### 3.1. Initial Cu Microstructure and Thermal Response

The three plating processes produced distinct Cu microstructures and thermal responses, as shown in Figure 2. The CG condition exhibited a coarse-grained morphology with an average grain size of 1.2 µm after the 100 °C, 1 h pre-bond anneal and remained largely unchanged after the 210 °C, 2 h bonding thermal cycle, with an average grain size of 1.3 µm. In contrast, both FG-L and FG-TD conditions retained fine-grained microstructures after the pre-bond anneal, with average grain sizes of 130 nm and 125 nm, respectively. These results establish three distinct microstructural states prior to Cu–Cu bonding. The two fine-grained conditions were differentiated by their response to the bonding thermal cycle. FG-L remained fine-grained, reaching an average post-bonding anneal grain size of 165 nm, whereas FG-TD underwent substantial recrystallization and coarsening to 412 nm. Thus, FG-L represents a thermally stable fine-grained Cu microstructure, while FG-TD represents a fine-grained starting state with pronounced thermally dynamic grain growth. This distinction is significant because fine-grained Cu possesses a high grain-boundary density and stored strain energy, while thermally activated grain growth can enhance grain-boundary migration and promote cross-interface microstructural evolution under a reduced bonding thermal budget [11,12,32]. The three conditions therefore provide a controlled basis for separating the effects of initial grain size, microstructural stability, and top/bottom grain-size asymmetry on the subsequent bonded-interface response.

### 3.2. Qualitative Morphology of the Bonded Interfaces

Representative bonded-interface morphologies for the five Cu microstructure combinations are shown in Figure 7. The symmetric CG/CG interface exhibited the least favorable morphology, with numerous planar interfacial voids, limited grain intermixing between the top and bottom Cu layers, and minimal grain penetration across the bond line. In contrast, the FG-L/FG-L interface exhibited a lower density of interfacial voids and greater grain continuity across the bond line while retaining a predominantly fine-grained structure following bonding. FG-TD/FG-TD also exhibited substantial overall intermixing, with grain growth concentrated near the bond line; however, regions of localized grain coarsening within the bottom Cu layer were associated with reduced cross-interface intermixing, indicating spatially nonuniform interface evolution.

The asymmetric interfaces exhibited more pronounced directional grain growth across the interface. In CG/FG-L, some interfacial voids remained; however, grain penetration from the CG side toward the FG-L side was more extensive than observed for CG/CG, resulting in greater cross-interface intermixing. This directional evolution is consistent with the grain-size asymmetry contributing to curvature-mediated grain-boundary migration across the bonded interface [24]. CG/FG-TD exhibited the highest qualitative degree of intermixing along the bond line and fewer interfacial voids. Although the post-bond grain size of FG-TD remained smaller than that of CG, the interface exhibited greater metallurgical continuity and fewer residual voids. This behavior is consistent with grain-size asymmetry, facilitating grain advancement across the interface and promoting bond-line elimination under the common thermal cycle.

Overall, the qualitative observations reveal a progression from the void-rich, weakly intermixed CG/CG interface to the more continuous FG-containing interfaces, with the strongest apparent intermixing observed for CG/FG-TD. Nevertheless, qualitative inspection alone cannot distinguish widespread bond-line bridging from localized deep grain penetration, nor can it quantify the residual void burden. The five combinations were therefore compared quantitatively using BIF, IVL, and normalized GPD in the following section.

### 3.3. Quantitative Bond IQ Analysis of the Bonded Interfaces

Quantitative Bond IQ analysis demonstrated that the three descriptors resolve different but coupled components of Cu–Cu interface evolution (Figure 7). CG/CG exhibited the weakest response, with an IVL of 5.4 nm, a BIF of 6.1%, and a normalized GPD of 0.20. The combination of high IVL, low BIF, and low GPD is consistent with the numerous planar voids, persistent bond line, and restricted cross-interface growth observed qualitatively. This behavior is consistent with comparatively low grain-boundary density of CG Cu, which provides fewer fast diffusion pathways and limits void shrinkage and grain-boundary migration under the applied thermal budget [17]. In addition, the residual planar voids along the CG/CG bond line may pin the interface-adjacent grain boundaries (Zener-type pinning [25,26]) and further restrain boundary migration and cross-interface grain growth; this interpretation was not verified experimentally. In contrast, all interfaces containing FG-Cu layer showed markedly lower IVL and higher BIF, indicating that the presence of a fine-grained layer promoted both interface closure and void reduction.

The symmetric fine-grained interfaces illustrate how microstructural stability influences bond line bridging, void elimination, and penetration. FG-L/FG-L yielded an IVL of 0.5 nm, a BIF of 21.9%, and a normalized GPD of 0.54, whereas FG-TD/FG-TD yielded an IVL of 1.2 nm, a BIF of 20.6%, and a normalized GPD of 0.41. For the present blanket thermocompression-bonded structure, FG-L/FG-L exhibited the more favorable quantitative response. The retained fine-grained FG-L structure may provide abundant grain-boundary diffusion pathways that facilitate void shrinkage, consistent with its lowest measured IVL, while its thermal stability may limit widespread coarsening. In contrast, FG-TD/FG-TD exhibited substantial recrystallization and grain growth in the vicinity of bond line; however, localized growth within the individual Cu layers, particularly within the bottom layer, may partially accommodate the available stored energy without contributing proportionally to cross-interface bridging. Consequently, the two symmetric interfaces achieved comparable BIF values through distinct microstructural pathways.

The asymmetric interfaces exhibited the most pronounced interfacial evolution but through distinctly different microstructural pathways. CG/FG-L exhibited an IVL of 1.1 nm, a BIF of 32.7%, and the highest normalized GPD of 0.89. This combination of moderate interface coverage and highest penetration is consistent with localized directional growth: a limited population of CG grains may advance deeply into the thermally stable FG-L layer at favorable interface sites, while resistance of FG-L to widespread coarsening restricts distributed lateral bridging. CG/FG-TD, in contrast, exhibited an IVL of 0.6 nm, the highest BIF of 54.5%, and a normalized GPD of 0.27. Its high BIF but modest GPD indicates distributed, relatively shallow intermixing. This behavior is consistent with two concurrent processes. First, grain-size asymmetry may promote widespread migration of coarse-grained boundaries toward the finer-grained FG-TD layer [16,24]. Second, thermally activated recrystallization and grain growth within FG-TD near the bond line may generate numerous short bridging segments. Together, these processes would increase interfacial coverage while contributing only modest grain penetration depths.

Across the five conditions, BIF generally increased as IVL decreased, indicating that progressive bond-line elimination was accompanied by a reduction in residual interfacial void content and an increase in structurally continuous Cu across the interface. Nevertheless, the non-monotonic relationship between BIF and normalized GPD shows that lateral interface elimination and penetration depth are complementary rather than interchangeable. CG/FG-TD favored broad, shallow intermixing and near-minimum void loading, whereas CG/FG-L favored spatially selective but substantially deeper grain advancement. The fine-grained side may provide a high density of fast grain-boundary diffusion pathways, while the CG/FG grain-size contrast may supply a curvature-related driving force for migration from the coarse-grained side toward the finer-grained side [16]. These probable mechanisms are consistent with the enhanced interfacial evolution of the FG-containing interfaces relative to CG/CG, which also produced different BIF–GPD signatures. Retaining BIF, IVL, and normalized GPD as independent descriptors is therefore essential for distinguishing distributed interface closure from localized penetration and defect removal. The mechanisms invoked in this section (fine-grain-assisted diffusion, curvature-mediated boundary migration, and recrystallization near the bond line) are interpretations that are consistent with, but not directly demonstrated by, the measured descriptors and grain sizes; the experimental observations are the BIF, IVL, GPD, and grain-size values themselves.

The reported descriptor values were obtained from a single representative, approximately 100 µm-wide stitched cross-sections for each condition. This cross-section was selected using criteria defined before the analysis, namely complete interface coverage, minimal sample-preparation artifacts, good registration of the SE and ion-channel images, and image quality suitable for reliable segmentation, and not because it showed the most favorable result. Two additional cross-sections were examined to verify qualitative consistency of the observed interfacial morphology. These sections were prepared from the same bonded pair and showed the same overall trend, although some interface segments were more void-rich and others more void-free (continuous); they were not included in the quantitative analysis. Together, the sections capture within-sample spatial variability and do not represent independent bonding runs, and the variation in the descriptor values among cross-sections was therefore not quantified. Accordingly, the reported descriptor values represent large-area interface measurements rather than means derived from independent replicates, and therefore no statistical error bars are reported. The relative performance should be interpreted within the context of the present blanket chip-level thermocompression-bonded structure. Patterned hybrid-bonding wafers may exhibit different qualitative and quantitative responses because Cu recess, dielectric confinement, local contact geometry, and spatial variations in bonding pressure can influence interface closure, void evolution, and grain growth behavior [10,27].

### 3.4. Implications, Limitations and Scope of the Framework

The present work establishes Bond IQ as an automated classical computer-vision framework for quantifying the degree of Cu–Cu grain intermixing from registered cross-sectional FIB-SEM images through three complementary descriptors: BIF for lateral interface bridging, IVL for residual void loading, and normalized GPD for cross-interface grain advancement. Beyond enabling objective comparison of bonding conditions, the measured descriptors are consistent with several proposed, but not directly verified, mechanisms of grain-growth behavior: fine-grained Cu may enhance atomic transport through its high grain-boundary density, grain-size asymmetry may promote curvature-mediated migration from the coarse-grained side toward the finer-grained side, and the thermal stability of FG-L versus the recrystallization response of FG-TD can shift the interface between localized deep penetration and distributed shallow bridging. Thus, Bond IQ combines quantitative metrology with materials-science interpretation of interface evolution, replacing subjective visual assessment with physically meaningful metrics for screening Cu microstructures and bonding conditions. The present ranking is specific to blanket chip-level thermocompression bonding and may differ in patterned hybrid-bonding structures containing Cu recess and dielectric confinement; future correlation with electrical resistance, bond strength, and reliability [10] is therefore required before the descriptors can serve as performance-qualification criteria. The descriptors reported here are microstructural continuity indicators; they have not been validated against mechanical (e.g., bond strength) or electrical (e.g., contact resistance) measurements and should not be read as direct measures of the functional quality or reliability of the bonds.

Sensitivity to analysis parameters. The descriptors depend on the segmentation settings and on the reconstructed bond line. IVL is mainly affected by void detection (Otsu thresholding) and by the ±0.5 µm analysis band. BIF and GPD are mainly affected by grain-boundary detection and by the bridge classification (including the 3-pixel minimum bridge width). All three descriptors are also affected by a shift or displacement of the reconstructed bond line, because this changes which voids fall inside the analysis band and which grains are classified as spanning the interface. A systematic parametric sensitivity analysis was not performed in this study. To limit this effect, the same processing settings were applied to all samples and the overlays were checked against the original images (Section 2.3), and the reported values should be compared only among datasets processed in the same way. A quantitative sensitivity analysis is recommended for future work.

Thermal exposure and stability. The metrics in this study describe the bond interface after a single bonding cycle (210 °C, 2 h) and do not demonstrate long-term thermal stability. Additional annealing or thermal cycling may change grain growth, residual stress, pore structure, and interface continuity [8], for example, through thermal-expansion mismatch between Cu and the surrounding materials [9,33] and through the stability of the structure under thermal exposure, which is an important design constraint in thermally loaded systems [34]. Future work should monitor BIF, IVL, and GPD after different thermal conditions and correlate the results with mechanical, electrical, and reliability performance.

## 4. Conclusions

This study establishes Bond IQ as an automated classical computer-vision framework for quantifying Cu–Cu grain intermixing through three complementary descriptors: BIF, IVL, and normalized GPD. Application to five blanket chip-level Cu microstructure combinations showed that interfacial evolution depends not only on initial grain size but also on microstructural stability and top/bottom grain-size asymmetry. CG/CG exhibited the highest void loading and the lowest bridging and penetration. CG/FG-TD produced the highest BIF (54.5%) and low IVL (0.6 nm), consistent with distributed, relatively shallow interface closure arising from grain-size-driven boundary migration coupled with local FG-TD recrystallization. In contrast, CG/FG-L produced the highest normalized GPD (0.89), indicating localized but deeper directional grain advancement, while FG-L/FG-L exhibited the lowest IVL (0.5 nm) and the most favorable symmetric-interface response. A central finding is that interfacial bridging, residual void loading, and grain penetration depth are not interchangeable indicators of bond quality; rather, they capture distinct and complementary aspects of Cu–Cu interfacial evolution. The proposed mechanisms, which are interpretations of the observations rather than directly verified results, involve fine-grain-assisted diffusion, curvature-mediated grain-boundary migration, and the different thermal responses of FG-L and FG-TD. The present ranking applies to blanket chip-level thermocompression bonding and may change in patterned hybrid-bonding structures containing Cu recess and dielectric confinement. The descriptors are microstructural continuity indicators and have not yet been validated against mechanical or electrical performance. Future work should correlate the descriptors with electrical resistance, mechanical strength, and reliability to establish application-specific acceptance criteria.

## Figures and Tables

**Figure 1:**
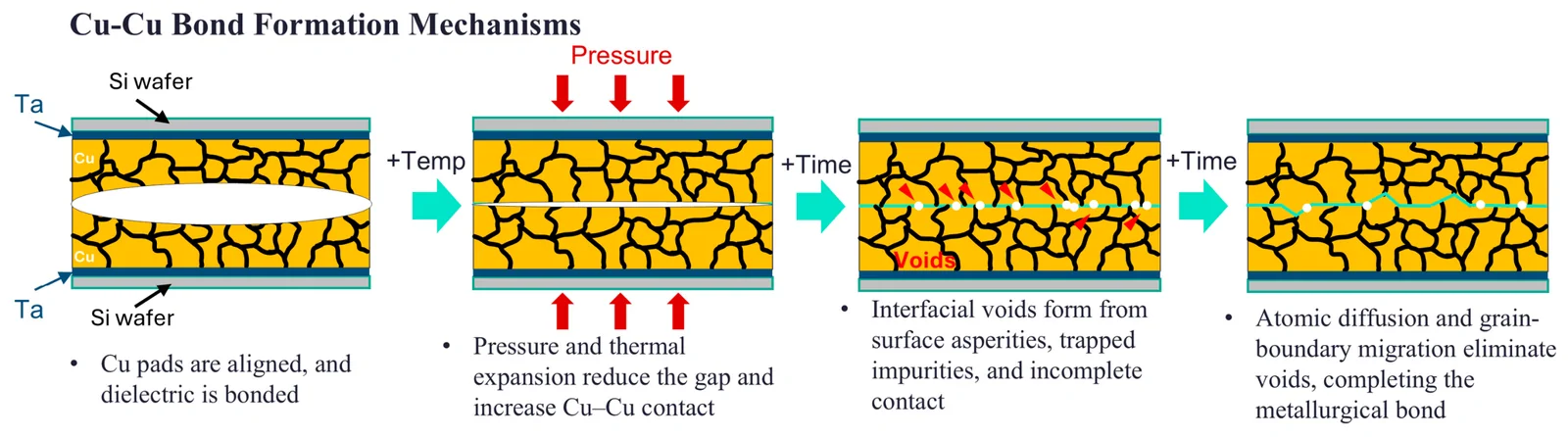
Schematic illustration of Cu–Cu bond formation during thermocompression bonding, adapted from Refs. [2,11,19,20,21], showing interfacial contact evolution under applied pressure and temperature, void formation, and subsequent void elimination through atomic diffusion and grain-boundary migration. Red arrows indicate the applied bonding pressure, and the Ta barrier layer (blue) lies between each Si wafer and the Cu film.

**Figure 2:**
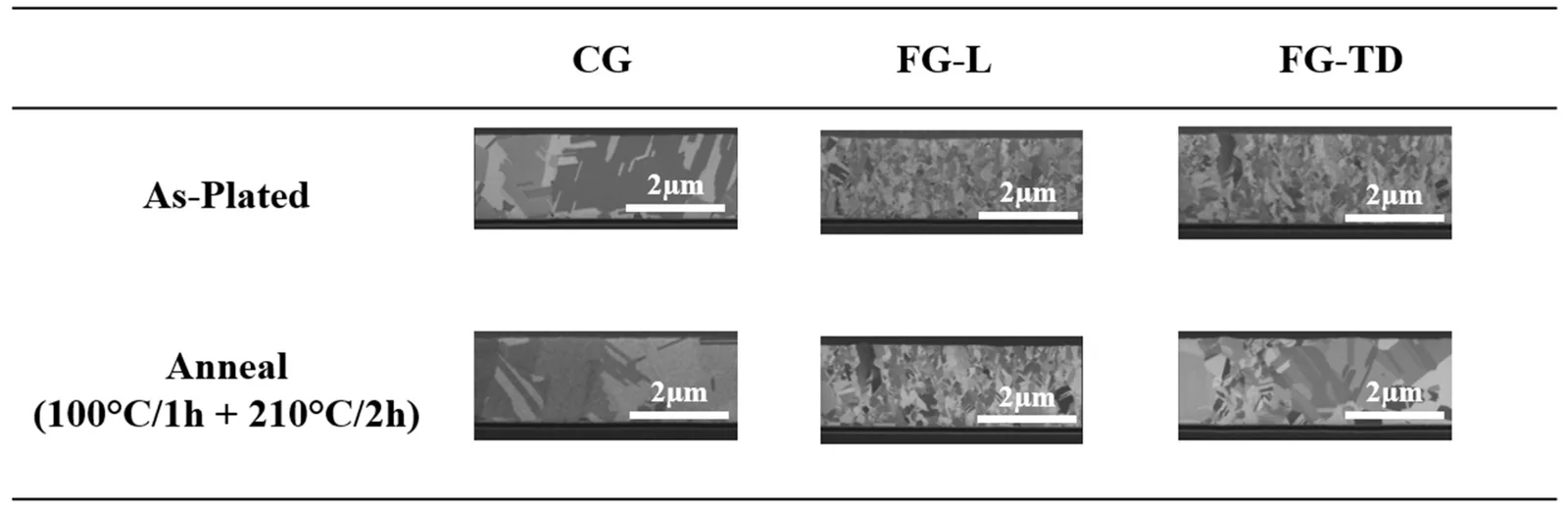
Representative plan-view Cu microstructures of the CG, FG-L, and FG-TD conditions before and after annealing.

**Figure 3:**
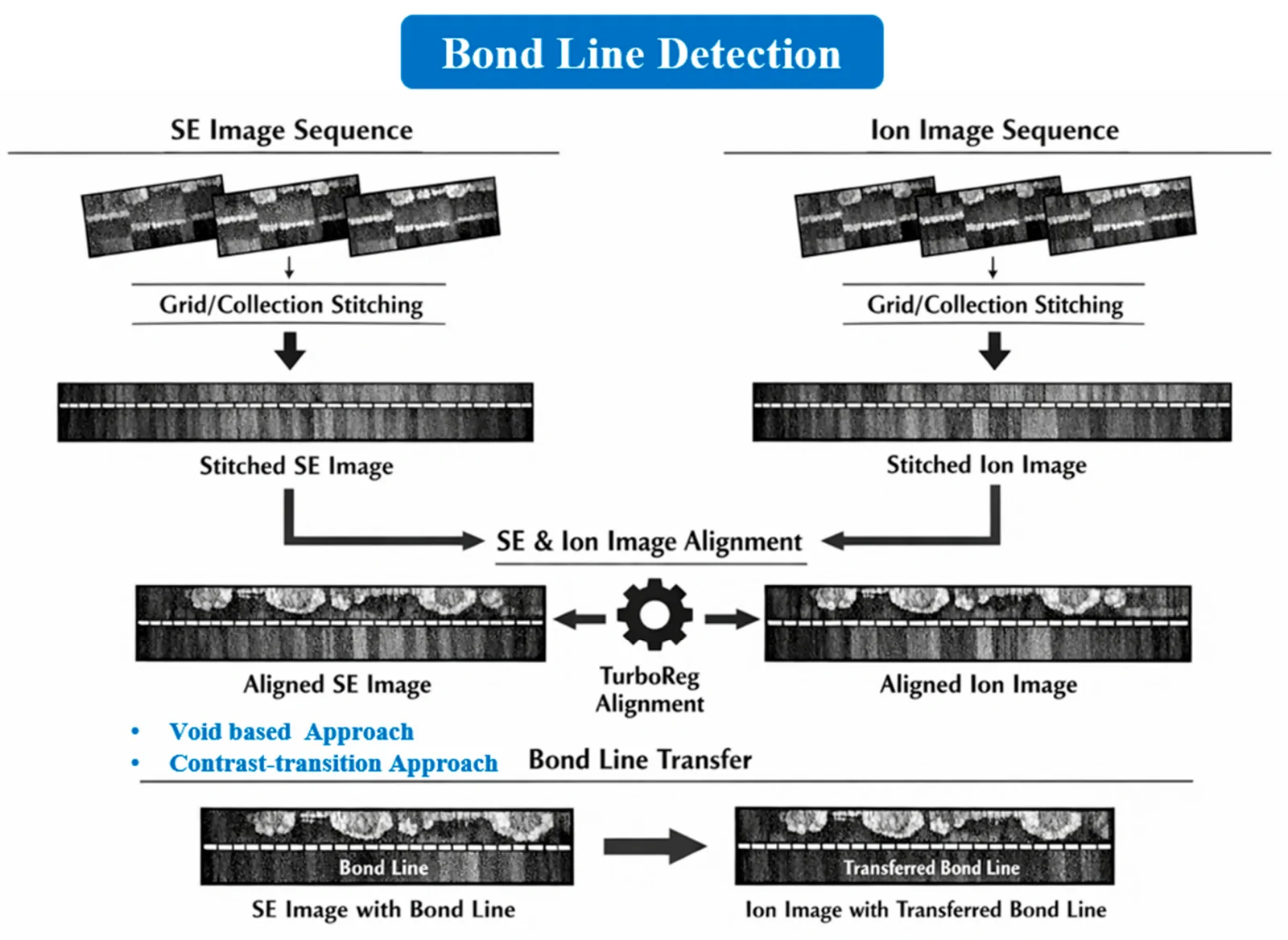
Bond-line detection workflow illustrating image stitching, SE–ion image alignment, and transfer of the detected bond line to the registered ion-channel image. Although Bond IQ supports both void-based and contrast-transition detection, the void-based method was used in this study: centers of visible interfacial voids in the SE image were used as reference points, and the bond line trajectory was interpolated across regions without visible voids.

**Figure 4:**
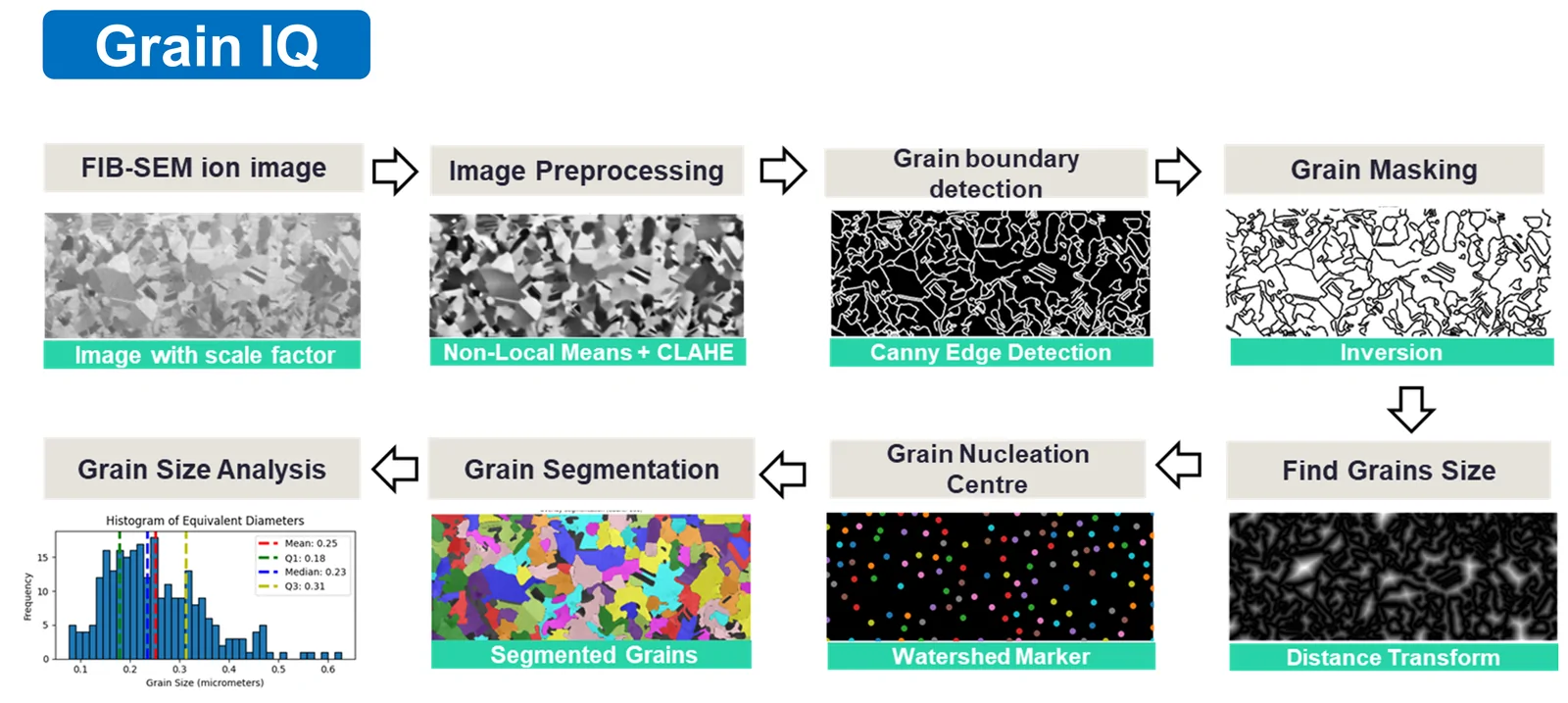
Workflow of the Grain IQ framework for automated grain segmentation and grain size measurement from FIB-SEM images.

**Figure 5:**
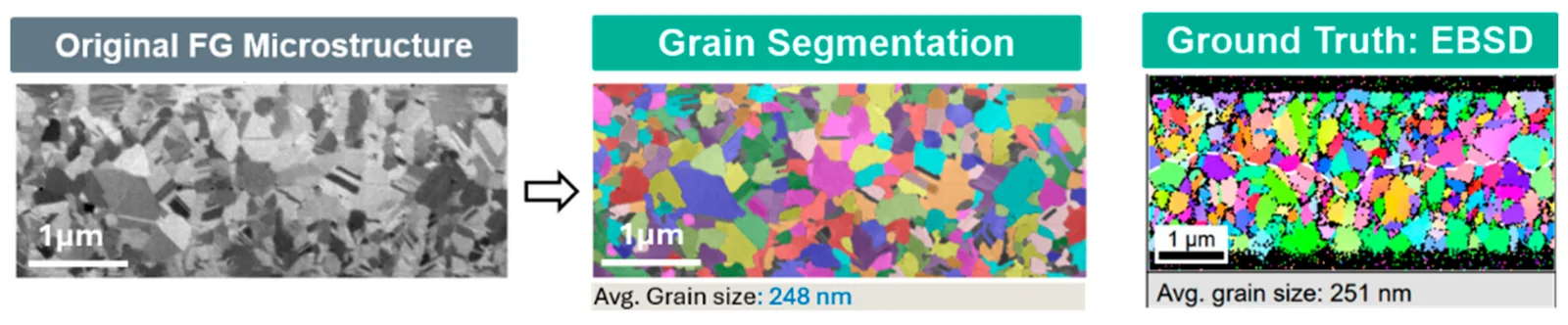
Representative validation of the Grain IQ grain-segmentation framework against EBSD using the same specimen and processing condition. For example, the average grain size measured by Grain IQ was 248 nm, compared with 251 nm from EBSD.

**Figure 6:**
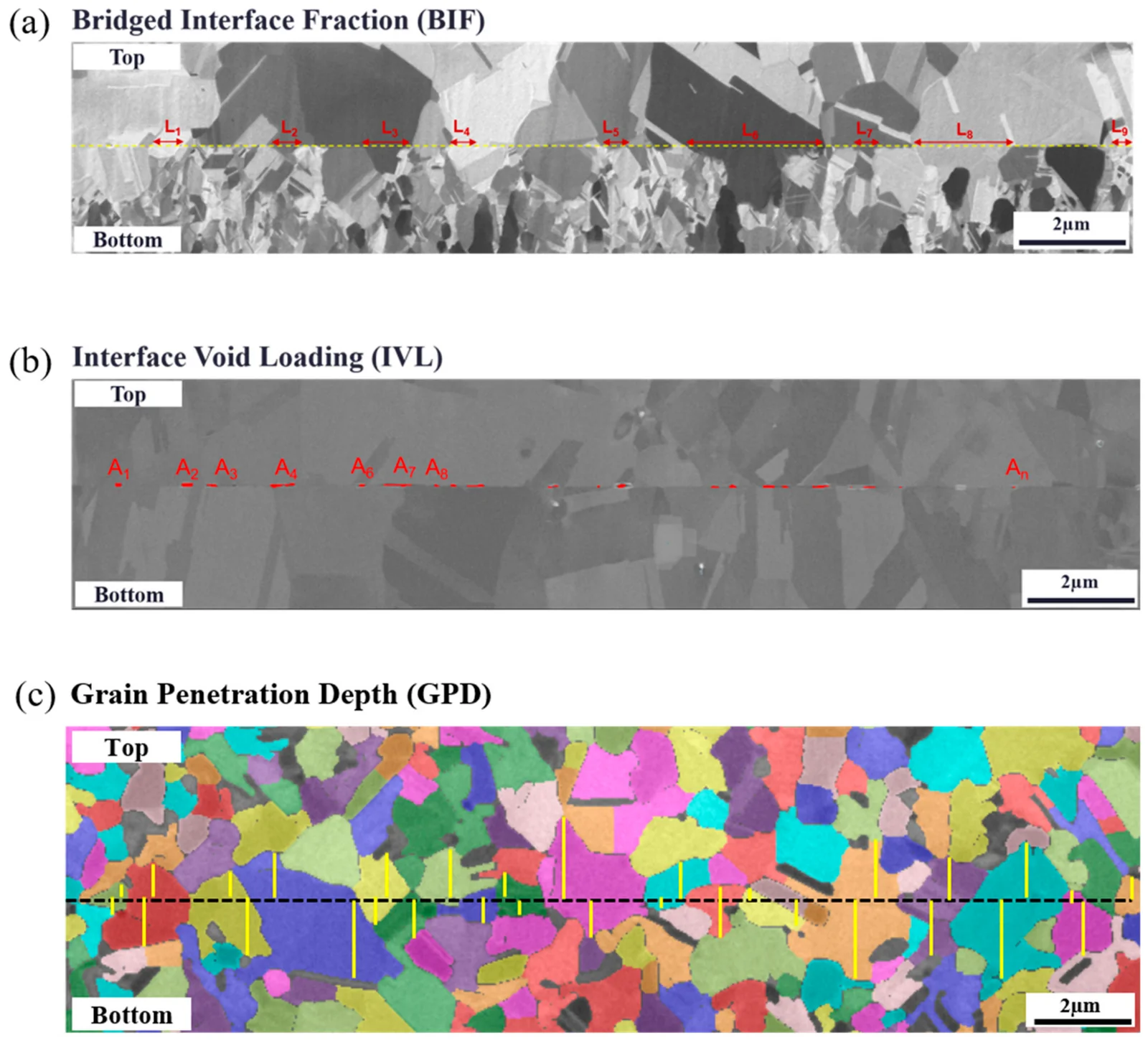
Representative illustration of the quantitative metrics extracted by Bond IQ for Cu–Cu bond characterization. (**a**) Bridged Interface Fraction (BIF), which characterizes interfacial contact continuity; the red double-headed arrows (L_1_–L_9_) mark the bridged segments along the bond line (yellow dashed line). (**b**) Interface Void Loading (IVL), which quantifies void coverage along the bond line; the red regions (A_1_–A_n_) mark the interfacial voids. (**c**) Grain Penetration Depth (GPD), which measures grain growth across the bonding interface; the yellow vertical lines indicate the penetration distance of grains spanning the bond line (black dashed line) into the opposing Cu layer. Scale bars: 2 µm.

**Figure 7:**
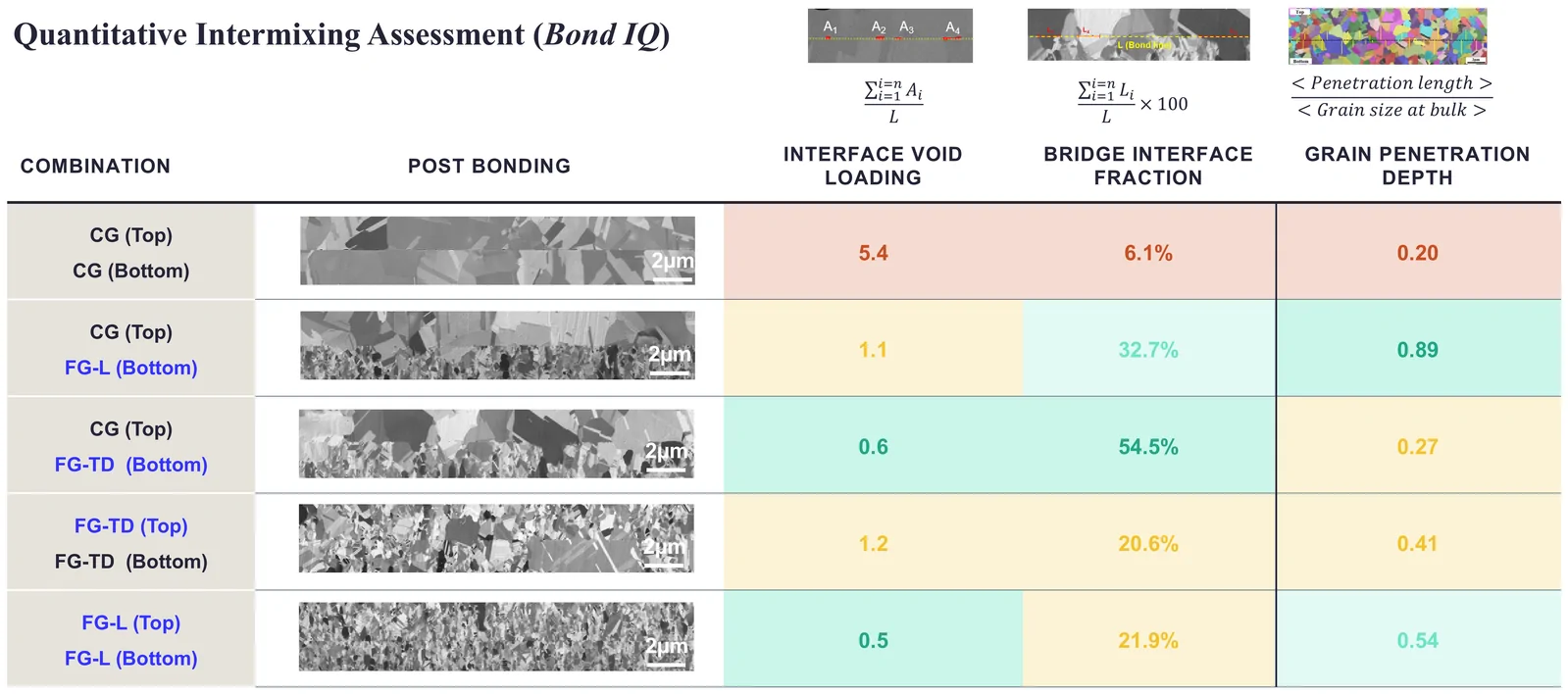
Bond IQ analysis results for the different Cu–Cu bonding combinations evaluated in this study. Bond quality and intermixing were quantified using IVL, BIF, and GPD, which assess interfacial voiding, bonded interface continuity, and grain penetration across the bonding interface, respectively.

**Table 1:** Process designations used in this study and the corresponding Cu microstructure classification.

Cu Type.	Processes	Description
CG	Process A	Coarse-grained Cu
FG-L	Process B	Fine-grained Cu with limited grain growth
FG-TD	Process C	Fine-grained Cu with thermally dynamic grain growth

## Data Availability

The data that support the findings of this study are available from the corresponding author upon reasonable request, subject to company policies and confidentiality restrictions.
